# Automatic Skin Cancer Detection Using Clinical Images: A Comprehensive Review

**DOI:** 10.3390/life13112123

**Published:** 2023-10-26

**Authors:** Sana Nazari, Rafael Garcia

**Affiliations:** Computer Vision and Robotics Group, University of Girona, 17003 Girona, Spain; rafael.garcia@udg.edu

**Keywords:** skin cancer detection, melanoma detection, automated diagnosis of pigmented skin lesions (PSLs), computer-aided diagnosis, literature review, clinical skin images

## Abstract

Skin cancer has become increasingly common over the past decade, with melanoma being the most aggressive type. Hence, early detection of skin cancer and melanoma is essential in dermatology. Computational methods can be a valuable tool for assisting dermatologists in identifying skin cancer. Most research in machine learning for skin cancer detection has focused on dermoscopy images due to the existence of larger image datasets. However, general practitioners typically do not have access to a dermoscope and must rely on naked-eye examinations or standard clinical images. By using standard, off-the-shelf cameras to detect high-risk moles, machine learning has also proven to be an effective tool. The objective of this paper is to provide a comprehensive review of image-processing techniques for skin cancer detection using clinical images. In this study, we evaluate 51 state-of-the-art articles that have used machine learning methods to detect skin cancer over the past decade, focusing on clinical datasets. Even though several studies have been conducted in this field, there are still few publicly available clinical datasets with sufficient data that can be used as a benchmark, especially when compared to the existing dermoscopy databases. In addition, we observed that the available artifact removal approaches are not quite adequate in some cases and may also have a negative impact on the models. Moreover, the majority of the reviewed articles are working with single-lesion images and do not consider typical mole patterns and temporal changes in the lesions of each patient.

## 1. Introduction

Skin cancers have been on the rise in recent decades, becoming a very significant public health issue [1]. Among the different types of skin cancer, melanoma is one of the deadliest types, including 80% of deaths from skin cancer [2]. In the past decade (2012–2022), the number of new invasive melanoma cases diagnosed annually increased by 31% [3]. However, early detection is vital to the possibility of effective treatment: the estimated five-year survival rate for patients whose melanoma is detected early is about 99 percent. This survival rate falls to 68 percent when the disease reaches the lymph nodes and 30 percent when the disease metastasizes to distant organs [3]. Taking these numbers into account, it is imperative to identify skin cancer and melanoma as early as possible.

Pigmented skin lesions (PSLs) represent a diverse spectrum of dermatological conditions characterized by an anomaly on the skin, presenting itself as a discolored spot due to the presence of various pigments. These lesions are of significant clinical importance, as they encompass both benign entities, such as moles and freckles, and malignant conditions, such as melanoma and non-melanoma skin cancer [4,5].

A common method for diagnosing PSLs is the use of dermoscopy, a non-invasive technique that exploits the use of a magnifying lens (a dermoscope) and liquid immersion to magnify submacroscopic structures [6]. It enhances the sensitivity of the naked-eye examinations in clinical practice [7]. However, an early-stage case of skin cancer may only receive an opinion from a non-specialist (e.g., a physician who is not trained in dermatology) with only standard cameras as the imaging method at hand. In such cases, an image from the lesion can be captured and sent to a dermatologist for examination. This method has proven to be as effective as physical patient diagnosis with a much faster speed [8]. In a study done by Brinker et al. [9], a group of 157 dermatologists (including 60% dermatology residents and junior physicians) performed significantly worse on dermoscopic images than on clinical (macroscopic) images of various skin lesions in detecting melanoma. Consequently, melanoma detection is largely dependent on the imaging type.

**Figure 1:**
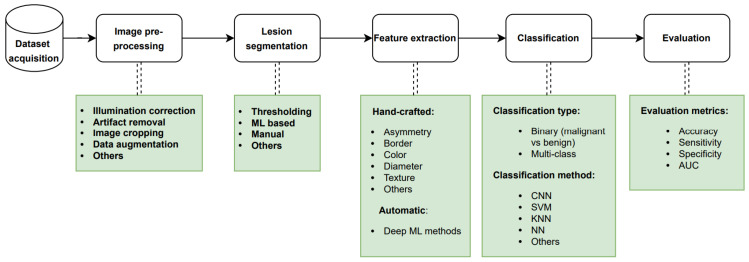
Standard pipeline for design and development of an automated skin cancer detection system. ML: machine learning, CNN: convolutional neural network, SVM: support vector machine, KNN: K-nearest neighbor, NN: neural network, AUC: area under the ROC curve.

In the past decades, there have been developments in computational methods for helping dermatologists diagnose skin cancer early. Computerized analysis of pigmented skin lesions is a growing field of research. Its main goal is to develop reliable automated tools to recognize skin cancer from images. Studies have shown that automated systems are capable of diagnosing melanoma under experimental conditions [10]. Moreover, computer-aided diagnosis (CAD) systems have the potential to prove useful as a backup for specialist diagnosis, reducing the risk of missed melanomas in highly selected patient populations [11]. Machine learning (ML) has evolved considerably over the past decade due to the availability of larger image databases and improvements in computer architecture. Advances in deep neural networks have also been a critical factor in making deep learning gradually supplant customary machine learning models for the detection of skin cancer. The conventional procedure employed for automated skin cancer detection involves a sequence of steps that include acquiring the image data, performing pre-processing, segmenting the pre-processed image, extracting the relevant features, and subsequently classifying the image based on the extracted features as depicted in Figure 1. The final step of the approach is the evaluation of the trained classifier using proper metrics. It should be noted that the segmentation and feature extraction steps may be skipped depending on the ML method employed.

### Motivation and Contribution

Melanoma is one of the most fatal cancers. This type of cancer has a high death rate and is typically detected in advanced stages, but research from the previous decade indicates that early diagnosis of malignant lesions can drastically lower patient mortality rates and survival rates. Many researchers have worked to use different methods of imaging and artificial intelligence to detect and diagnose these malignancies. Also, in order to address the issues related to the identification and diagnosis of these cancers, these researchers have put forth a number of unique and modified conventional methodologies.

However, while finding numerous evaluations on the identification of skin cancer using artificial intelligence, we were unable to locate a thorough analysis of the diagnosis of skin cancer using clinical (macroscopic) images and machine learning methods. Furthermore, we could not find any work that presented all the available clinical datasets in a comprehensive manner. We have conducted a comparison of the presented survey with other recent reviews in Table 1. Our presented survey was subjected to a comparative analysis with existing reviews, based on various criteria such as the year scope, imaging modality type, and coverage, as well as the major tasks involved in the automated skin cancer detection pipeline, as depicted in Figure 1. Furthermore, we conducted an assessment to verify if the papers elaborated on technical details or solely focused on the results, and examined if all available clinical datasets had been reviewed thoroughly.

Stiff et al. [12] comprehensively reviewed the literature on clinical images, with a primary focus on the application of convolutional neural networks (CNNs) for image classification. However, their review lacked a complete overview of the available clinical datasets. In contrast, Wen et al. [13] provided a thorough survey of all available datasets, encompassing various image modalities, including clinical data. Nevertheless, Wen’s work only focused on the datasets themselves. On the other hand, Manhas et al. [14] conducted a general review of works done on automated skin cancer detection. However, their research did not provide a summary of available clinical datasets and instead focused mostly on the challenges researchers face in the field. Similarly, Bhatt et al. [15] and Jones and Rehg [16] conducted general surveys of state-of-the-art techniques for skin cancer detection, with a main emphasis on machine learning methods for classification. Consequently, they failed to present a detailed review of works done on clinical datasets. Finally, Haggenmüller et al. [17] solely concentrated on research of melanoma detection using convolutional neural networks. Although, their review did not include a comprehensive evaluation of segmentation and feature extraction tasks. Meanwhile, Dildar et al. [18] mainly centered their survey on neural network-based skin cancer detection studies.

In order to analyze the work of the researchers of the reviewed papers in terms of the pipelines they implemented and the results they offered, we attempt to present the most significant research articles that have used clinical image data for the detection, segmentation, and classification of skin cancers in this research paper. The contribution of this study is to provide a critical and thorough analysis of several artificial intelligence techniques and their use in the diagnosis of cancerous lesions on the skin using clinical data based on performance evaluation metrics. Our objective is to provide a review article that serves as a comprehensive reference for researchers interested in acquiring knowledge and conducting systematic skin cancer detection using clinical skin images.

In this paper, we review the research on automatic skin cancer detection approaches using machine learning in the past decade with a special focus on clinical images. It is important to acknowledge that melanoma, being the most aggressive form of skin cancer, was the primary focus of the majority of the papers we reviewed. Consequently, a greater emphasis is given to the diagnosis and detection of melanoma throughout our article. In the following section, we will briefly describe skin cancer types and their causes, as well as different imaging modalities used to monitor skin lesions. In Section 3, a detailed discussion of the search scope used for selecting the papers will be presented. Next, we will discuss various datasets used in the state-of-the-art papers. Later, in Section 4, we will review the selected articles in terms of pre-processing, image segmentation, feature extraction, change detection, and other diagnostic and classification methods. Lastly, the Section 5 of the article highlights the numerous key findings from this review.

## 2. Background

### 2.1. Skin Cancer

Unrepaired DNA damage in the external layer of the skin (epidermis) causes mutations that trigger uncontrolled growth of abnormal cells, which may give rise to skin cancer. Malignant tumors develop when the skin cells multiply excessively because of these mutations. Sunlight’s ultraviolet (UV) exposure and tanning beds’ UV rays are the two major risk factors for malignant tumors and skin cancer. The main types of skin cancer are basal cell carcinoma (BCC), squamous cell carcinoma (SCC), melanoma, and Merkel cell carcinoma (MCC) [2]. Other forms of skin cancer include Kaposi’s sarcoma, dermatofibrosarcoma protuberans (DFSP), cutaneous T-cell lymphoma (CTCL), sebaceous gland carcinoma, and atypical fibroxanthoma (AFX). However, these conditions are characterized by their rarity and comparatively lower associated risks when contrasted with the more frequently encountered types of skin cancer [19,20].

Melanoma is a type of skin cancer that develops when melanocytes (the cells that give the skin pigment) start to grow out of control. Although melanoma is the least common type of skin cancer, it is the most dangerous one. Additionally, it has a higher chance of spreading to other parts of the body unless it is diagnosed and treated early.

A benign skin tumor is called a mole (nevus) that develops from melanocytes. Moles are very common in the general population. Almost all moles (nevi) are not harmful, but some types can raise the risk of melanoma.

### 2.2. Imaging Methods

There are various types of imaging devices for skin cancer detection. The most common equipment used to investigate the characteristics of pigmented skin lesions is the dermoscope, which is used with a conventional digital camera. Dermoscopic images display subsurface microstructures of the epidermis and upper dermis. However, these devices are not widely available for public use [21,22].

On the other hand, conventional digital cameras with a spatial resolution (without the dermoscope) are commonly used by non-dermatologists such as primary healthcare professionals. Images taken by these devices are called macroscopic or clinical images. This type of image is often unevenly illuminated [23].

**Figure 2:**
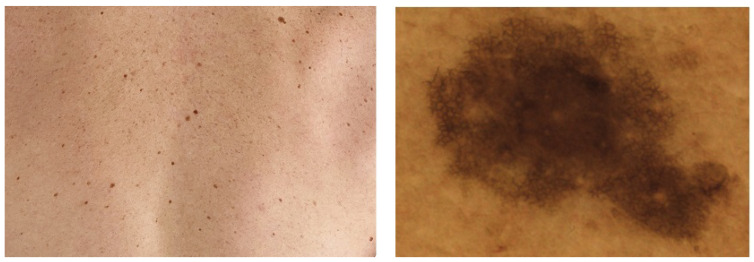
Different types of skin images. (**Left**): regional image, (**Right**): individual image.

Independently from the imaging device used, a PSL image can include one single lesion or an area of multiple lesions. Images covering multiple lesions are called regional or wide-field images. In Figure 2, you can see samples of both image types. In this article, we focus on work conducted on individual macroscopic images.

### 2.3. Automated Diagnosis of Skin Cancer

Over the past decade, various types of skin images, including dermoscopic and clinical images, have been collected with the purpose of being used as training data for the automatic diagnosis of skin cancer, specifically melanoma. Numerous machine learning methods have been implemented and proposed for the effective detection of different types of skin cancer, and several of these methods require segmentation and feature extraction techniques.

## 3. Search Criteria

This section addresses the identification of the scope of the reviewed articles. The process for identifying the scope is based on the imaging technique, machine learning models employed, pre-processing procedure, segmentation techniques utilized, features extracted, and performance evaluation metrics.

In this review paper, we aim to select different studies on skin cancer and its diagnosis using machine learning techniques. The publications in this review were acquired from the following databases: IEEE Xplore, Science Direct (Elsevier), Springer Link, PubMed, Arxiv, and Google Scholar.

**Figure 3:**
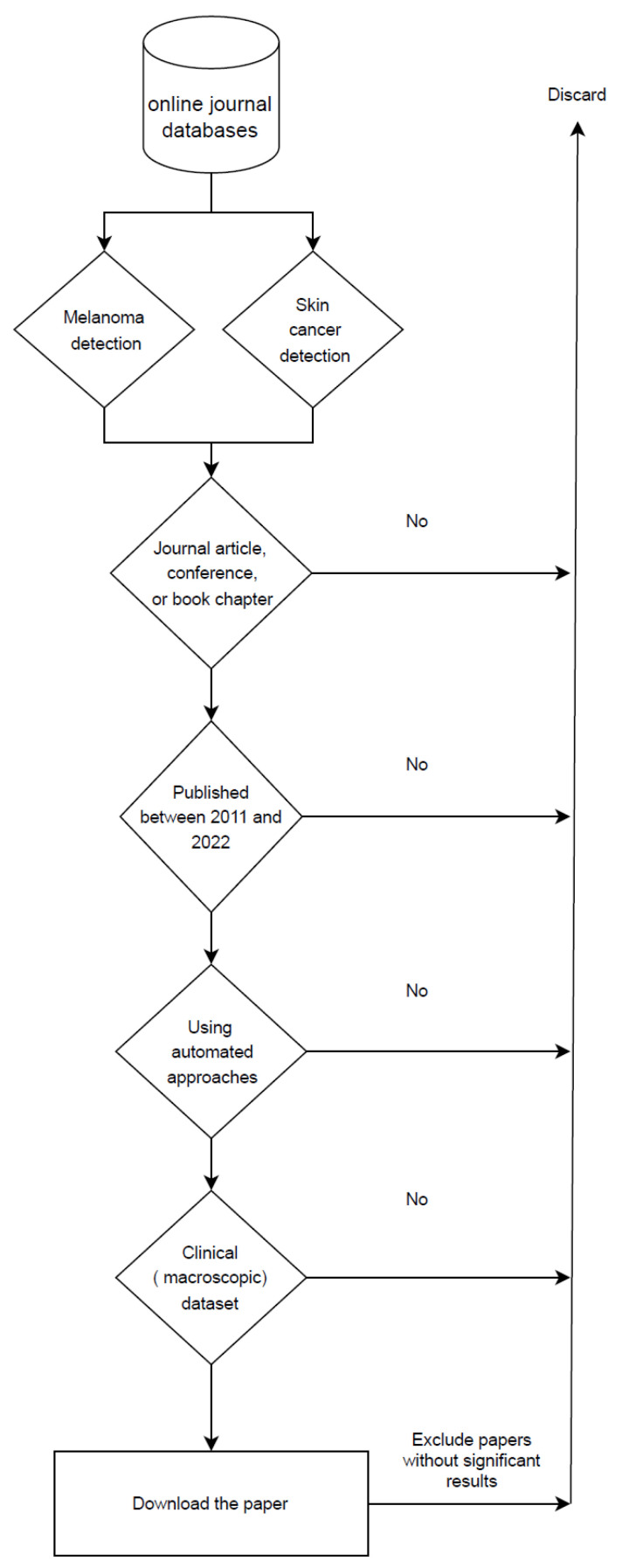
Flowchart of search criteria for research articles in this review paper.

Regarding the methodology, we first started our search by using relevant keywords such as skin cancer, automated skin cancer detection, artificial intelligence in dermatology, melanoma detection, skin cancer detection with machine learning, and machine learning. We gathered articles from journals, conferences, and books that focused on the automated detection of skin lesion tasks from 2011 to 2022, excluding earlier publications. From the papers published in 2022, only those that were out by the time of compilation of this paper are included. Also, we removed articles that used datasets that were not entirely clinical. Furthermore, we excluded articles that failed to produce acceptable results compared to the other available research in the same scope. In other words, papers that neither improved the outcomes of prior research nor introduced novel methodologies. Following a thorough examination of these papers, only 51 were chosen based on our research criteria, as illustrated in Figure 3.

## 4. Literature Review

### 4.1. Datasets

There are several publicly available datasets of clinical skin images that have been used by different teams over the past decade. The most frequently used datasets are DermQuest and MED-NODE. The papers reviewed in this article present error metrics that are not comparable since they are measured using different datasets. For this reason, Table 2 provides a brief overview of these different datasets. All the details regarding the current public datasets of clinical images are explained in Appendix A.

### 4.2. Pre-Processing

Images taken by cameras usually contain undesired artifacts that make the segmentation process more difficult. The pre-processing of a clinical image attempts to correct some of these irregularities caused during image acquisition, such as illumination artifacts, presence of hairs, or ruler markings. Pre-processing is essential for a proper analysis at this stage so that the algorithms will behave correctly in subsequent analysis [74].

Mainly, the reviewed papers used four different types of pre-processing tasks with different methods. We list these tasks and the associated papers in Table 3. In addition, the pre-processing methods used by state-of-the-art papers are divided into five groups as explained in the subsections below.

#### 4.2.1. Illumination Correction (Shading Attenuation)

As described above, the collected images may contain illumination artifacts, and if they are used directly as segmentation input, shading and lesion borders may be confused. Therefore, shading is attenuated in the input image before image segmentation. The reviewed papers have used various approaches to remove shading and reflections from their datasets. We will explain them in more detail below.

Cavalcanti and Scharcanski [44] proposed a data-driven method for shading attenuation in HSV color space that was also used by Amelard et al. [47], Cavalcanti et al. [65] and Amelard et al. [49].

In the work of Giotis et al. [54], illumination effects are eliminated by smoothing out steep gradients in the saturation and value channels, using the HSV color space. Other works [53,55,56,63] followed the same approach.

Amelard et al. [46] proposed a multi-stage illumination correction algorithm for removing reflective artifacts from images. The same authors, in another work [51], used the previous approach but proposed a novel multistage illumination modeling algorithm (MSIM) to enhance illumination variation in clinical images. This new approach was based on computing an initial estimate of the illumination map of the photograph using a Monte Carlo non-parametric modeling strategy. Glaister et al. [66] and Amelard et al. [48] authors also used MSIM for reflection reduction.

Marín et al. [59] removed reflective artifacts with a thresholding algorithm and in-painting operation that was proposed in Barata et al. [75] A median filter with the mask size calculated from Equation (1), combined with a shadow reduction method in HSV color space, was proposed by Ramezani et al. [43] for shading attenuation. Mask size ‘*n*’ in Equation (1) is determined for a W × H image and the floor function rounds down the result to the nearest integer. The authors also used K-means to remove non-uniform illumination caused by the flashlight used to acquire the images.

(1) $$n=floor(5\cdot \sqrt{\left(W/768)\cdot (H/512\right)})$$

#### 4.2.2. Artifact Removal

Artifacts in macroscopic images are defined as noise, skin lines, body hair, and skin stains, among other effects.

DullRazor [76], a popular tool for hair artifact removal, was used by several researchers [42,54,59] to remove hair effects on skin images. Huang et al. [71] proposed a hair segmentation and deletion method that exploited matched filtering and region-growing algorithms. Two-dimensional (2D) matched filters are shape-specified pixel patterns that convolve with a grayscale image to find similar patterns within the image. Then, Huang et al. [71] implemented DullRazor and their proposed method on their own dataset and compared both methods. They concluded that their method had a lower false-hair detection rate (by 58%) than DullRazor. However, this has not been demonstrated on a public dataset.

Ramezani et al. [43] applied a button hat morphological transformation followed by morphological opening to remove thick hairs from the images. Oliveira et al. [39] applied an anisotropic diffusion filter [76] to remove hair artifacts. Morphological closing operations with interpolation of pixels of hair with neighboring pixels were performed on the image to eliminate hair by Sagar and Saini [53]. These authors also applied a median filter for noise reduction. Al-Hammouri et al. [50] used the same approach for noise reduction.

Huang et al. [71] applied multi-scale curvilinear matched filtering, followed by hysteresis thresholding [77] for detecting hair. Subsequently, they detected intersections of hair using linear discriminant analysis (LDA) [78]. Additionally, Huang et al. [71] compared their method with DullRazor visually and claimed that their method performed better on their dataset with a hair detection rate of 0.81 and a false hair detection rate of 0.18.

The Gaussian filter has been widely used in several works [27,41,54,55,56,64] to reduce noise in skin images. Jafari et al. [67] used an edge-preserving method called guided filter [79] in their work [67] to remove the presence of artifacts. In later work, Jafari et al. [56] and Giotis et al. [54,56,63] reduced the additional remaining noise effects by applying the Kuwahara smoothing filter [80].

- Comparative Analysis

**Figure 4:**
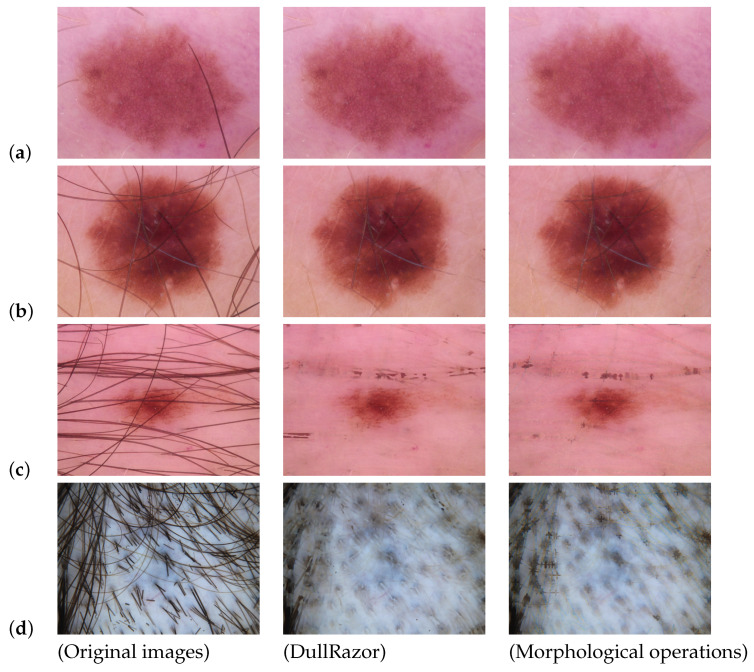
Comparing different hair removal approaches. Original images: Original images from the HAM10000 dataset [82]; DullRazor: Images corrected with DullRazor method; Morphological operations: Images corrected with morphological operations. In image (**a**), DullRazor was able to successfully eliminate the artifact while the morphological-based algorithm left some traces of the artifact in the image. In image (**b**), both methods fail to remove the artifact from the lesion area. In image (**c**), DullRazor removed artifacts from the lesion area, but there are hair traces present in the skin area. However, the morphological-based method failed the task. In image (**d**), we can see that attempting to remove the artifact does not provide an informative output image.

We tested the two most commonly used hair removal approaches on some skin lesion images. The first approach is DullRazor, and the second one is based on morphological Black Hat transforms combined with thresholding. The results can be seen in Figure 4. As we see from the processed images, both methods can remove small hair artifacts and improve the images. However, there are some downsides to using each of them. DullRazor may remove most of the hair without damaging the lesion’s features, but in some situations, it has limited accuracy, and some of the artifacts are still visible on the lesion. The same issue appears with the morphological operations. Also, the morphological-based approach smoothed out the pixels of the image, and because of that, some of the lesion features were rendered invisible. In addition, there may be some images that provide zero information to the diagnosis due to the excessive amount of hair artifacts (see Figure 4) present on the lesion. In these cases, hair removal algorithms may not be beneficial. Therefore, the best strategy to deal with these types of images is to consider them as outlier data and remove them from the dataset. It is also worth noting that in recent work, some researchers [81] augmented their datasets by adding hair, and others did not perform any artifact removal pre-processing.

All in all, artifact removal algorithms can improve the automated diagnosis of PSLs if they are used carefully. Otherwise, they may have a negative impact on the training phase of the ML algorithms.

#### 4.2.3. Image Cropping

One of the important factors in training CNN-based classification models is to have all the images the same size. For this purpose, researchers often use cropping, resizing, and re-scaling methods to unify dataset shapes. Some papers reviewed implemented these methods on their datasets.

#### 4.2.4. Data Augmentation

Data augmentation is useful to improve the performance of machine learning models by creating new and different examples to train datasets. If a dataset in a machine learning model is rich in informative features and sufficient in size, the model performs better and achieves higher accuracy. Data augmentation techniques enable machine learning models to be more robust by creating variations that the model may meet in the real world. Moreover, augmentation reduces the chance of model over-fitting. Also, skin image datasets are often imbalanced because the number of melanoma cases is much lower than other skin diseases. In this scenario, data augmentation can be used to balance the datasets. In the papers reviewed here, researchers have used various methods to augment their datasets.

Some papers [28,29,32,36,51,55,62,64,73] used standard data augmentation methods such as cropping, rotation, flipping, and scaling for augmenting their data. Others [28,35,36,40,51,62] used different tools like guided filter, Gaussian low-pass filter, blur filter, noise addition, motion blur, histogram equalization, Poisson, salt-and-pepper, speckle, and JPEG compression to add noise to the dataset. In addition, color and brightness changes were applied by Glaister et al. [51] and Pacheco and Krohling [35]. Up-sampling, down-sampling, and oversampling were other methods of data augmentation that were used by Pacheco and Krohling [35] and Castro et al. [31].

#### 4.2.5. Other Methods

Some authors performed different types of pre-processing on their datasets, and we will review them in this section. Aggarwal and Papay [38] darkened the skin area in images with lighter skin color using fast contrastive unpaired translation (FastCUT) [83] and reported that darkening the skin area can improve the performance of the classifier. In another study, Al-Hammouri et al. [50] applied contrast enhancement and histogram equalization [84] to improve the segmentation task. Castro et al. [31] also applied the mixup extrapolation balancing (MUPEB) [85,86] algorithms to balance their dataset. In addition, Krohling et al. [32] and Castro et al. [31] used two different approaches to balance their dataset. First, they applied a weighted loss function, and then they proposed another method based on the mutation operator from the differential evolution algorithm, a technique they called differential evolution (DE).

### 4.3. Segmentation

Image segmentation is the process of dividing an image into multiple sections or pixel sets (also referred to as image objects). Segmentation makes the image easier to analyze [87]. Lesion extraction from an image under analysis can be made easier through segmentation. However, it should be noted that the segmentation of skin images is a very difficult task that may require some pre-processing or/and post-processing.

Our reviewed papers have proposed many different approaches for segmentation. Among all of them, Otsu’s method [88] and K-means clustering were the most widely used.

The standard Otsu standard method was used by Mukherjee et al. [60], Al-Hammouri et al. [50], and Biasi et al. [64]. Ramezani et al. [43] proposed a threshold-based segmentation method using Otsu’s method combined with the mean value of lesion and healthy skin distribution peaks, healthy skin Gaussian distribution, and lowest height between the lesion and healthy skin distribution on the histogram. The single-channel images that contain factors that determine a lesion’s border (color, illumination, and texture) are obtained first. Ramezani et al. [43] evaluated their work by presenting the segmented images to dermatologists. According to the authors, the physicians’ diagnosis indicated that these methods were accurate in determining the borders and extent of lesions, with 100% accuracy in determining the extent of lesions. However, in such subjective tasks, the reliability of the presented accuracy remains ambiguous. In another investigation, Do et al. [68] used fast skin detection followed by Otsu’s method and minimum spanning tree (MST) [89] composed for segmenting the images. However, they did not present any evaluation of their segmentation results. A color channel-based method, which combined a modified effective two-dimensional Otsu method with an edge detection algorithm was proposed by Sagar and Saini [53]. In this case, the authors reported that in the task of detecting the correct lesion from an input digital image, their algorithm is approximately 93.7% accurate.

On the other hand, the standard K-means (with K = 2) clustering algorithm was used as a segmentation method by many authors [27,41,54,55,56,58,63]. Jafari et al. [56,63] performed K-means (with K = 2) clustering in HSV color space. In another study, a combination of Otsu thresholding and K-means was proposed by Munia et al. [58], reporting an accuracy of 89.07% and an AUC of 0.91 on their dataset. The authors of Khan et al. [41] also proposed an improved version of K-means (with K = 2) with an accuracy of 94%. Lastly, in Shihab et al. [27], an adaptive threshold image segmentation with K-means clustering was proposed. However, these accuracy metrics are not comparable since every method uses a different dataset.

Among other methods, Chan–Vese’s active contour method [90] was implemented by Shalu [59] for segmenting images. Furthermore, Cavalcanti et al. [65] proposed a new segmentation method that is a combination of fast independent component analysis (FastICA) [91] lesion localization and Chan–Vese’s [90] level-set boundary detection. Cavalcanti et al. [65] used the segmentation error ϵ for evaluating the performance of their method and reported a segmentation error of 16.55%. In the same report, they claimed that this error was the lowest compared to the other state-of-the-art works that were using the same evaluation method [65]. Another method using Chan–Vese’s active contour algorithm and some morphological operations was advanced by Oliveira et al. [39]. Afterward, segmented images were visually assessed by dermatologists. However, Oliveira et al. [39] did not report any quantitative results.

Do et al. [52] suggested a segmentation method based on skin detection [92] and a combination of Otsu’s method with minimum spanning tree (MST) [89] followed by a border localization algorithm. The authors used true detection rate (TDR), a measure of how many pixels are correctly classified as lesions in an image, as their evaluation method. Do et al. [52] achieved a TDR of 80.09%, which outperformed the Otsu and MST algorithms on the same dataset. Moreover, Sabouri and GholamHosseini [37] used a CNN for border detection by classifying image patches and reported a Jaccard index of 86.67% for their data. In other reports, GANs with a U-Net generator were used to detect and segment pigmented lesions by Udrea and Mitra [33] and reported 91.40% accuracy. However, their accuracy metric was based on the percentage of correctly segmented lesions. In a later study, Araujo et al. [45] used a U-net with four subsampling layers that had been proposed by Ronneberger et al. [93] for biomedical image segmentation. They reported a sensitivity of 92%, a specificity of 98%, and an accuracy of 95%. Jafari et al. [67] implemented pixel classification using a CNN and obtained 98.70% accuracy. Also, Jadhav et al. [73] classified image patches to the lesion and healthy classes by applying an SVM with the cubic kernel to features extracted by a CNN. This research achieved an accuracy of 95% and a Jaccard index of 89%. On the other hand, Devi et al. [61] proposed a fuzzy C-means clustering method for segmenting clinical images, reporting 95.69% accuracy, 90.02% sensitivity, and 99.15% specificity. Glaister et al. [66] advanced a segmentation algorithm based on joint statistical texture distinctiveness (TD) that uses K-means and reported an accuracy of 98.30% on their dataset.

Interactive object recognition [94] was another method that was used by Sabouri et al. [42] for segmentation. Moussa et al. [72] proposed a new segmentation method based on thresholding, edge detection, and considering one connected component of the mask. Later, the graph-cut segmentation technique for macroscopic images was proposed by Pillay and Viriri [23]. On the other hand, a novel segmentation algorithm, called synthesis and convergence of intermediate decaying omnigradients (SCIDOG), was developed by Albert [62] and claimed to have more robustness against noise, artifacts, and low contrast. In another investigation by Cavalcanti and Scharcanski [44], a 3-channel image representation was generated and utilized to differentiate between the lesion and healthy skin areas. Also, Glaister et al. [51] applied the statistical region merging (SRM) [95] algorithm to their dataset for segmenting their images. Finally, Amelard et al. [46,47,48,49] and Marín et al. [70] used manual segmentation for their work. Unfortunately, none of the methods mentioned in this paragraph presented any quantitative or qualitative evaluation of their segmentation approaches. Therefore, we cannot evaluate the performance of the proposed approaches.

Post-processing is the implementation of any technique to enhance and improve the segmented image. Many of the state-of-the-art papers used post-processing methods to enhance their segmentation results. Among them, the most popular methods applied were morphological operations(opening, closing, and hole-filling) and Gaussian filtering. Araujo et al. [45] implemented two more steps in their pre-processing task. After the morphological closing of the segmented area, they removed the artifacts from the lesion by a region mapping operation. Afterwards, they used a hole-filling algorithm.

### 4.4. Feature Extraction

In machine learning, feature extraction and feature selection are considered crucial steps. In state-of-the-art papers working on skin lesion diagnosis, authors have historically used a variety of features. For example, most papers used hand-crafted features based on the ABCD rule of dermatology. Asymmetry, border irregularity, color, diameter, and texture are the most commonly used hand-crafted features among the reviewed articles. Feature selection techniques are used in some cases to reduce the size of the feature set. Some studies, on the contrary, used deep neural networks and left feature extraction to be achieved by CNNs.

The ABCD acronym for melanoma diagnosis was established in 1985 to provide an early detection criterion for malignant melanoma among healthcare professionals. Asymmetry, border irregularity, color variation, and diameter greater than 6 mm have been recognized as the main criteria for the evaluation of pigmented cutaneous lesions, which may require further evaluation by a specialist [96].

ABCD features are still being used in recent papers [23,44,56]. Moussa et al. [72] extracted geometric features based on asymmetry, border irregularity, and diameter. On the other hand, some researchers [43,52,58,62,68] combined the ABCD criteria with texture features. The total number of extracted features by each paper is shown in Table 4. In the next subsections, the methods used for extracting hand-crafted features from the reviewed papers will be summarized.

#### 4.4.1. Asymmetry Features

An asymmetrical structure is more likely to occur in malignant than in benign lesions [97]. Do et al. [52,68] calculated asymmetry features by identifying the major and minor axes of the lesion region as proposed by Celebi et al. [98]. In the work of Oliveira et al. [39] an axis based on the longest diagonal vector defined by Euclidean distance [99] of the lesion was used to divide the area of the lesion into two sections. Later, lesion borders were tracked with the Moor–Neighbour tracking algorithm [100] in Jafari et al. [56] for determining asymmetry features. In addition, central moments of the lesion were quantified by Green’s theorem [101] for determining asymmetry features by Albert [62]. In another work, Ramezani et al. [43] used the lesion’s center of gravity and inertial moments to identify asymmetry features.

On the other hand, Munia et al. [58] calculated solidity, variances of distance, and major/minor axis length as asymmetry features. Also, features such as solidity, extent, equivalent diameter, and circularity, among others, were extracted as asymmetry features in other works [44,46,48,49,51]. Amelard et al. [46,48,49] proposed high-level intuitive features (HLIFs) for asymmetry descriptors with respect to color and shape irregularities and combined them with features extracted in [44] to create their feature set.

Pillay and Viriri [23] calculated the asymmetry index (AI) as follows:

(2) $$AI=\frac{\Delta A}{A}\times 100$$

where (*A*) is the area of the total image, and (ΔA) is the area of the difference between the total image and lesion area.

Finally, Moussa et al. [72] calculated the asymmetry index (AI), as shown in Equation (3):

(3) $$AI=\frac{IA}{LA}$$

where (IA) is the intersection between the mirrored segmented image and segmented image, and (LA) is the original lesion. The authors established that a lesion with an AI smaller than 0.7 should be considered a melanoma.

#### 4.4.2. Border Features

Benign skin lesions tend to have smoother borders in comparison to malignant ones. A melanoma lesion, in most cases, has a notched or almost indistinguishable border indicating ongoing growth or spread of cancer. Do et al. [52,68] determined the irregularity of the border by considering shape features such as compactness, solidity, convexity, and variance of distance from border points to the lesion’s centroid, following the approach proposed by Celebi et al. [98]. They also proposed features for border irregularity in a procedure they called border fitting. In another article, Ramezani et al. [43] extracted 34 features for border irregularity and categorized them into sets based on the area and perimeter of the lesion, irregularity index, best-fit ellipse, convex hull, gradient, fractal geometry, area, two perimeters, four features based on the radius [102], border irregularity indices [103], compactness index [104], Heywood circularity index, mean curvature [105], best-fit ellipse indices [106], bulkiness index [107], bending energy, area, perimeter of the convex hull, convexity index [108], indentation and protrusion index, and fractal dimensions [109].

On the other hand, by using the inflection point descriptor (for measuring small irregularities in the border) and the vector product descriptor (for measuring substantial irregularities in the border), Oliveira et al. [39] identified the number of peaks, valleys, and straight lines in the border.

In addition, some authors [56,58,62] defined a convex hull around the lesion’s mask and calculated the error of the convex hull area as a border irregularity feature (*B*) according to the formula in Equation (4):

(4) $$B=\frac{Area\;of\;convex\;hull-area\;of\;lesion^{\prime }s\;mask}{Area\;of\;lesion^{\prime }s\;mask}$$

where Albert [62] and Pillay and Viriri [23] quantified the compact index (CI) of border irregularity, as shown in Equation (5):

(5) $$CI=\frac{P^{2}}{4\pi A}\times 100$$

where *P* is the perimeter of the lesion boundary, and *A* is the lesion area.

Albert [62] also calculated the error of a perimeter structured by the convex hull, where the lesion perimeter is considered as ground truth, as a third irregularity metric. The formulation of this error is presented in Equation (6):

(6) $$Error=\frac{C-P}{P}$$

where *C* is the convex hull perimeter, and *P* corresponds to the lesion perimeter.

Moreover, in the article by Moussa et al. [72], the researchers calculated the circularity index (CRC), as presented in Equation (7):

(7) $$CRC=\frac{4PA}{P}$$

where *A* is the area of the lesion, and *P* is the perimeter of the lesion. They also calculated the adjusted irregularity index (AIrA) as another border feature:

(8) $$AIrA=\frac{A}{P}$$

where *A* is the area of the lesion, and *P* is the perimeter of the lesion.

On the other hand, the average gradient magnitude of the pixels in the lesion extended rim and variance of the gradient magnitude of the pixels in the lesion extended rim [110] were extracted in the three color channels by Cavalcanti and Scharcanski [44]. The lesion rim irregularity was characterized by the ABCD rule by dividing the rim into eight symmetric regions: R=1,…,8. For each channel, the average gradient magnitudes of the extended rim pixels $\mu_{R,i}\left(R=1,\ldots ,8\right)$ were computed. From these, they calculated six more features based on the average and variance of the 8 $\mu_{R,i}$ values in each one of the three channels. Ramezani et al. [43] also applied the same approach.

Amelard et al. [46,47,48] proposed the use of high-level intuitive features (HLIFs) based on fine and coarse irregularities to define border descriptors. Amelard et al. [47] combined their features with the features extracted in Cavalcanti and Scharcanski [44] to create their final feature set.

#### 4.4.3. Color Features

In the papers reviewed here, color features were extracted in multiple color spaces, such as HSV, YCbCr, RGB, grayscale, BGR, and 1976 CIE L.a.b. (CIELAB). Also, the majority of researchers computed statistics such as minimum, maximum, mean, standard deviation, variance, skewness, entropy, normalized standard deviation, ratio of mean, and range of values from color channels of different color spaces as color features [39,41,42,43,44,51,52,54,56,58,59,60,68].

A malignant skin lesion might be black, blue-gray, red, white, light brown, or dark brown in hue. Each of the mentioned colors in the segmented image is worth one point, according to the method proposed by Pillay and Viriri [23] malignant lesions have three or more colors, whereas benign lesions exhibit two or fewer. Moreover, to reach one point, each present color must be greater than or equal to the individually stated threshold values. In this method, they used six different shades of each color mentioned above based on their RGB values and calculated their points as color features. However, the RGB color space is non-linear and discontinuous, which makes changes in color hue hard to follow. In addition, color hue is easily affected by illumination changes. Hence, color tracking and analysis is a complex task in the RGB color space and may cause false diagnosis in skin cancer detection. In the papers reviewed here, color features were extracted in multiple color spaces, such as HSV, YCbCr, RGB, grayscale, BGR, and 1976 CIE L.a.b. (CIELAB). Also, the majority of researchers computed statistics such as minimum, maximum, mean, standard deviation, variance, skewness, entropy, normalized standard deviation, the ratio of mean, and the range of values from color channels of different color spaces as color features [39,41,42,43,44,51,52,54,56,58,59,60,68].

A malignant skin lesion might be black, blue-gray, red, white, light brown, or dark brown in hue. Each of the mentioned colors in the segmented image is worth one point, according to the method proposed by Pillay and Viriri [23] malignant lesions have three or more colors, whereas benign lesions exhibit two or fewer. Moreover, to reach one point, each present color must be greater than or equal to the individually stated threshold values. In this method, they used six different shades of each color mentioned above based on their RGB values and calculated their points as color features. However, the RGB color space is non-linear and discontinuous, which makes changes in color hue hard to follow. In addition, color hue is easily affected by illumination changes. Hence, color tracking and analysis is a complex task in the RGB color space and may cause false diagnosis in skin cancer detection.

Alternatively, to capture color variation from the skin lesion, Do et al. [52,68] used the information from the histogram of pixel values. The same authors also proposed a novel descriptor to quantify color variation in skin lesions, and the color triangle feature, which is based on another article by Albert [62] that extracted the arithmetic mean, geometric mean, harmonic mean, and every tenth percentile (including min 0 and max 100) across three color spaces (color metrics were calculated across three color spaces: BGR, HSV, and 1976 CIELAB). Additionally, covariance was calculated for each color space. Later, these measures were generated for two sets of pixels: the closed lesion contour pixel set and the complementary set. Corresponding to the skin around the lesion, color metrics were generated with pixel-wise localization for the lesion region and the surrounding skin area. On the other hand, Choudhury et al. [69] used the color histogram to plot intensity distribution over pixels in skin images for capturing color features. High-level intuitive features (HLIFs) for color variation were proposed by Amelard et al. in [48]. They combined their color features with features extracted in Cavalcanti and Scharcanski [44]. Finally, Jafari et al. [63] defined and extracted three color feature descriptors, namely color variations, spatial color distribution, and intensity and color value.

#### 4.4.4. Diameter

In skin lesions, the diameter of the lesion is critical, as melanoma presents with rapid growth. This will result in a bigger diameter than the typical common moles. Moussa et al. [72] determined the diameter by calculating the smallest circle that could contain the lesion and then computing its diameter in pixels. The diameters were then divided by ten because of the large numbers they had. Their findings indicated that a malignant mole typically has an index greater than 15 or a diameter larger than 150 pixels. In another work by Pillay and Viriri [23], the authors classified a lesion as melanoma if the diameter is larger than or equal to 6 mm. They used Equation (9) to convert the major axis diameter of the segmented region of interest image to a millimeter scale:

(9) $$DM=\frac{Major\;axis\;length\times 25.4}{20\times dpi}$$

where dpi is the dots per inch, which is equal to 96.

Lastly, lesion diameter features were calculated in Ramezani et al. [43] from the best-fit ellipse diameter, major diameter, and maximum distance between two non-adjacent points on the lesion border.

#### 4.4.5. Texture Features

The gray-level co-occurrence matrix (GLCM) [111,112] of the grayscale channel is employed by many authors [41,42,43,50,52,58,60,62,68,69] to extract texture features. Hence, GLCM-based texture feature extraction is one of the most common approaches. Albert [62] also extracted co-occurrence matrix metrics from color images. In addition, the gray-level run length matrix (GLRLM) [113] was used by Mukherjee et al. [60].

On the other hand, Do et al. [68] captured edge information of lesions by applying Canny edge detection. Then, the authors normalized and counted the number of edge pixels of the lesion area. Afterwards, this number was used as a texture feature. Khan et al. [41] and Do et al. [52] used local binary pattern (LBP) [114] to obtain texture features of skin lesions. A learning approach, color image analysis learning vector quantization (CIA-LVQ) [115], was used by Giotis et al. [54]. In another work, fractal dimensions were computed from the dataset by using a box-counting method (BCM) in order to extract texture properties of the skin lesion by Oliveira et al. [39]. In addition, the histogram of oriented gradients (HOG) was employed as a textural descriptor by Choudhury et al. [69]. Finally, texture features were collected from the maximum, minimum, mean, and variance of the intensities of the pixels inside the lesion segment by Cavalcanti and Scharcanski [44] and Glaister et al. [51].

#### 4.4.6. Other Features

Some of the reviewed papers extracted features other than the ABCD and texture rule as their feature set. In this section, we are going to explain the details of these other extracted features.

Khan et al. [41] extracted multi-view local features based on the detected interest points. In this work, the interest points were determined by the difference of Gaussians (DoG) detector [116]. Yao et al. [57] also extracted the same interest points from images using the DoG detector to create their feature set. They also generated RGB color features, a scale-invariant feature transform (SIFT), and a local binary pattern (LBP) to further explain the discovered interest points.

Pacheco and Krohling [35] used the clinical features of the patients. Later, Castro et al. [31] combined features extracted from images by a CNN model with the clinical information of patients. In a later study [29], Pacheco and Krohling [35] proposed an attention-based mechanism to combine features extracted by CNN with patient clinical data. They named their proposed approach metadata processing block (MetaBlock). They reported that MetaBlock can be a better feature combination method compared to simple concatenation methods. In a recent study, Lima and Krohling [30] applied the same approach to their model.

Munia et al. [58] defined the complexity of the affected region by extracting a set of non-linear features. These extracted features are approximate entropy, sample entropy, and Hurst component. The approximate entropy and sample entropy values were used to determine the degree of irregularity in the image pixel patterns. Hurst components quantify the extent to which previous image pixel information is stored in subsequent pixels. On the other hand, a CNN model was implemented in the work of Jadhav et al. [73] to extract features. Yang et al. [26] also applied a pre-trained ResNet-50 to their model to extract deep features. Lastly, the total dermoscopy score (TDS) was calculated based on ABCD features and used as another feature by Pillay and Viriri [23] and Al-Hammouri et al. [50]. TDS is a semi-quantitative scoring system based on ABCD features that was proposed by dermatologists. In this method, separate scores for asymmetry, border, color, and dermoscopic structures are multiplied by weight factors and then summed. TDS values between 4.74 and 5.45 indicate suspicious lesions, and TDS values over 5.45 indicate melanoma [117]. In Table 5, weight factors and score ranges for each feature are shown. Note that some authors changed the weight factors in their work.

The total dermoscopy score is calculated as below:

(10) TDS=A·1.3+B·0.1+C·0.5+D·0.5

#### 4.4.7. Feature Selection and Normalization

Extracting all the possible features can result in large feature sets that may cause over-fitting or increase the model run-time during training. Because of that, authors often try to reduce the size of their feature set by choosing the most informative features. Feature selection is the procedure used to find a subset from the extracted feature sets with fewer features such that it maximizes the relevance between the subset and the original set. This relevance is characterized in terms of mutual information (MI). Articles by Do et al. [52,68] used the normalized mutual information feature selection (NMIFS) [118] method to select their features. They also proposed a novel criterion for feature selection that takes the feature coordinate into consideration while evaluating the goodness of features. It should be noted that this is only relevant when the lesion is centered in the image, which is normally the case. Afterward, Do et al. [68] used a transformation method called average neighborhood margin (ANM) maximization. In a different approach, Ramezani et al. [43] reduced the number of their features using principal component analysis (PCA).

In general, the extracted features may fall within different ranges. Therefore, classification performance drastically improves after feature scaling and normalizing. For this reason, researchers often apply a zero-mean normalization before passing the feature descriptors to the classifier. Most authors [40,43,44,50,51,68] used the z-score conversion method for normalizing their features.

### 4.5. Other Diagnosis Criteria

Some dermatologists proposed the expansion of the ABCD criteria to include an E for evolving (i.e., lesion change over time). An evolving lesion is a mole that has changed in size, shape, symptoms (e.g., itching, tenderness), surface (e.g., bleeding), or color. There is substantial evidence that monitoring the evolution of lesions following the ABCD rule facilitates the physician’s recognition of melanomas at an earlier stage. Additionally, evolution can recognize the dynamic nature of skin malignancy [96]. The process of measuring lesion evolution is called change detection. Variations in the size of a skin lesion over time can be a symptom of skin cancer. If a lesion expands in size over time, this may signal that it is cancerous and should be checked by a dermatologist. Automated technologies identify changes in skin lesions more consistently, sensitively, and efficiently, which can lead to earlier detection and better outcomes for patients. However, it is important to note that automated systems should be used as an aid to, and not a replacement for, the expertise of dermatologists. Currently, there are very few studies conducted on automated skin lesion change detection using clinical images.

Korotkov et al. [34] presented a novel scanner for detecting changes in pigmented skin lesions, which can be indicative of skin cancer. The system uses high-resolution cameras, computer vision algorithms, and machine learning techniques to capture images of the patient’s skin and analyze them for changes over time. The authors tested the scanner on a group of patients with multiple pigmented lesions and found that it was able to accurately detect changes in the lesions over time, with performance comparable to that of trained dermatologists. This system has the potential as a valuable tool for skin cancer screening and early detection, potentially improving patient outcomes and saving lives. Later, Korotkov et al. [119] proposed a computer-aided matching technique to improve the accuracy of skin lesion matching in total body photography, overcoming the difficulties of effectively recognizing and matching non-melanoma or non-pigmented lesions. The suggested method involved extracting specific features from photos and using them to match lesions between images acquired at various intervals. The authors evaluated the performance of the proposed method on a ground-truth dataset of more than 73,000 lesions and reported a high level of accuracy in matching lesions, with a sensitivity of 92.3% and a specificity of 99.5%. Their methodology provides a reliable and efficient method for matching skin lesions, which can aid in the early identification and monitoring of skin cancer. Automatically detecting growth in skin lesions can be a very effective task in malignancy diagnosis since the naked eye may not be as precise in identifying very small changes.

In another recent work, Soenksen et al. [120] proposed a computer-aided system to detect and classify suspicious skin lesions from wide-field images. For this purpose, they formed a dataset of 38,282 images containing 15,244 non-dermoscopic images. First, they used a blob detection algorithm to distinguish lesions and skin from other objects present in the images. Then, they applied single-lesion classification (patient-independent) using VGG16 architecture and ugly duckling scoring (patient-dependent) methods to evaluate PSLs. As a result of the ugly duckling criteria, each lesion has a likelihood of being suspicious based on its disparity to all other visible lesions in the wide-field image of the body. Finally, the outputs of both methods were combined to generate a single suspiciousness score for each lesion. They achieved 90.3% sensitivity and 89.9% specificity.

### 4.6. Lesion Classification

Detecting skin cancer with computer-aided systems involves pre-processing images, segmenting images, extracting features from the segmented images, and finally classifying each image (and thus each lesion) into binary or multiple classes. In the classification step, the extracted descriptors are used to interpret and provide information about PSLs. In other words, a classification model is developed based on samples from the training set to be used by one or more classifiers. For the learning process, each sample includes features extracted from an image and corresponding class values, which are given to the classifier as inputs. Hence, the performance of the model depends on the features and on the classifier. In addition, comparing classification strategies is only relevant when performed on the same dataset and the same set of features [74]. A summary of ML methods used by reviewed state-of-the-art articles is detailed in Table 6.

In recent years, CNNs have been some of the most preferred approaches for image classification in the reviewed papers. CNNs are neural networks (NNs) in which convolutional and pooling layers are sequentially combined, followed by fully connected layers at the end, similar to multi-layer neural networks. Using CNNs, images can be classified more accurately by automatically extracting local features from images. Moreover, CNNs are often easier to train than fully connected networks and have a lower number of hyper-parameters to tune. Some papers have trained custom CNN models for classifying skin lesion images [27,36,37,55]. Jafari et al. [67] implemented two identical CNN models with two convolutional layers to analyze the local texture and general structure of skin images. Then, they concatenated them into a fully connected layer. Additionally, Pomponiu et al. [40] and Jadhav et al. [73] used CNNs for extracting features from images. Pomponiu et al. [40] used a CNN with five convolutional layers followed by two fully connected ones while Jadhav et al. [73] implemented a three-layer CNN.

Some authors fine-tuned pre-trained CNNs (trained on ImageNet [121]) for their classification tasks. Fujisawa et al. [28] and Pacheco and Krohling [35] applied GoogleNet [122]. Pacheco and Krohling [35], Castro et al. [31] and Krohling et al. [32] used ResNet50 [123] on images combined with clinical features in their work. Later, Pacheco and Krohling [29] implemented an EfficientNet-B4 pre-trained on ImageNet. Han et al. [25] fine-tuned ResNet-152 model as their classifier. The same authors, in later work [24] trained a CNN to classify images into 134 different classes. They also added a binary (malignant/benign) discrimination task and a treatment prediction task to their classifier. In another study, Albert [62] proposed the PECK algorithm that merges a deep convolutional neural network (inception v3) with a support vector Machine and random forest classifiers. Aggarwal and Papay [38] used Inception-Resnet-V2 pre-trained with the ImageNet to classify BCC and melanoma. Biasi et al. [64] also implemented an AlexNet architecture pre-trained on ImageNet. Marín et al. [70] used a custom artificial neural network (ANN) used for the classification task. Lastly, Al-Hammouri et al. [50] used an extreme learning machine (ELM) that is a feed-forward neural network with three main layers (input, hidden, and output layers) and compared its performance with SVM, KNN (K = 5), and RF.

On the other hand, when using hand-crafted features, support vector machines (SVMs) have been the most popular machine learning method for classifying skin lesions. Amelard et al. [47] and Jafari et al. [56] applied linear SVMs to their models and Amelard et al. [47] implemented a linear soft-margin support vector machine. In addition, SVMs with radial basis function kernels were used by Ramezani et al. [43] and Sabouri et al. [42] as classifiers. An SVM model with the histogram intersection kernel was used as the classifier in Oliveira et al. [39]. Moreover, Jadhav et al. [73] had a cubic SVM classifier, and Do et al. [52] applied four hierarchical SVMs on four different feature sets. Finally, an SVM classifier was implemented in [68] (no kernel was specified). Additionally, Yang et al. [26] implemented a weighted SVM to classify multiple classes and updated the SVM weights based on the complexity level of each class.

Alternatively, Cavalcanti and Scharcanski [44] applied two ML models: A simple K-nearest neighbor (KNN) and a hybrid classifier built from a KNN followed by a decision tree (DT). This hybrid classifier proved to be able to reduce the number of false negatives in the binary classification of skin lesions. Later, Moussa et al. [72] applied a KNN for binary classification. In another work, the performance of five different classifiers: KNN (K = 10), multi-layer perceptron (MLP), random forest (RF), SVM (with a radial basis function kernel), and naïve Bayes based on different sets of features were evaluated by Sabouri et al. [42]. Munia et al. [58] also compared SVM (linear kernel), KNN (K = 20), DT, and RF classification achievements in their work. In another research, Shalu [59] made a comparison between DT, naïve Bayes, and KNN. The authors of [52] applied KNN on LBP features. Besides these, Pillay and Viriri [23] classified their skin lesions with total dermoscopy score (a semi-quantitative scoring system proposed by Nachbar et al. [97] based on ABCD features for each lesion), SVM, and KNN. Finally, KNN, DT, naïve Bayes, and SVM were compared in Khan et al. [41].

Moreover, Mukherjee et al. [60] used an MLP with the swarm optimizer for classification. Also, Giotis and Petkov [124] proposed a cluster-based adoptive metric (CLAM) classifier that was later used again by the same authors in [124]. In addition to that, Yao et al. [57] implemented a special common dictionary learning method, which was proposed and compared with K-means on different feature descriptors. Lastly, Lima and Krohling [30] used a pooling-based vision transformer (PiT) [125] architecture (pit_s_distilled_224) to classify their dataset into multiple classes.

### 4.7. Classification Results

In this section, we will discuss the results of the classification task of all the reviewed articles. Note that some of the articles did not have a classification section. Therefore, no results are reported from them. Furthermore, to be able to make a fair comparison between the classification performances of two or more works, they must have performed the classification task on the same datasets. In our case, most of the articles had different sets of images. Hence, we first present the results only for comparable papers in Table 7. In addition, some authors reported multiple sets of results for different ML methods or feature sets. Therefore, we only extracted the best outcome from each reference.

Results for the other articles that had similar datasets are grouped into different tables. In Table 7, it can be observed that the classification results for papers that used the MED-NODE dataset. Moreover, references that reported their findings based on the Dermofit dataset are shown in Table 8. In addition, since the authors that proposed HLIF also used a similar dataset, their classification results are grouped in Table 9. The best numbers are bolded in all tables. Also, the studies conducted on the PAD-USEF-20 dataset are grouped in Table 10. However, because the papers in Table 11 have worked on different datasets, we cannot report which paper has the best performance in comparison.

From Table 7, we can see that the work of Devi et al. [61] had the best performance on the MED-NODE dataset. Article Mukherjee et al. [36] has reported an accuracy of 90.58% on the Dermofit dataset, but they did not report sensitivity or specificity. And as we know, for evaluating melanoma detection, accuracy is not a sufficient metric, and sensitivity is the most important measurement. Accuracy should only be used when having a balanced dataset, and the value of false positives and false negatives is almost the same. None of these conditions are met in this case.

In Table 10, we include studies conducted on the PAD-USEF-20 dataset that were collected by the PAD mobile app [35]. Pacheco and Krohling [35] used a total of 1612 images and classified them into 6 classes. Later work, Castro et al. [31] used a total of 2057 images and divided them into cancer and non-cancer classes. Then, they combined the CNN model with patients’ clinical information. The authors reported a balanced accuracy of 92.39% and a sensitivity of 94.28% for distinguishing cancerous lesions from benign lesions. However, they reported a specificity of only 35.67%, which is not remarkable. In a later study, the same authors Krohling et al. [32] used the same dataset and the same CNN architecture with fewer clinical features to identify cancerous lesions. They were able to achieve a balanced accuracy of 85.5% and a sensitivity of 96.42%. Moreover, Pacheco and Krohling [29] used different methods to combine patients’ clinical data with features extracted by CNNs and reported that combining metadata with deep features can improve the performance of the classifier but the impact depends on the combination method. They also tested their approach using a dermoscopic dataset and concluded that applying patient clinical data resulted in a much larger improvement when the dataset contained clinical images. In recent work, Lima and Krohling [30] used transformers architecture as their classifier and demonstrated that a PiT model can outperform the other CNN models tested in the article. In all of their investigations, they reported that using patient clinical data in combination with features extracted by CNNs improved the classification results.

Additionally, Amelard et al. [46] has reported various metrics for the HLIF papers. In Table 11, it can be seen that they had both the highest specificity and accuracy on Amelard et al. [47] and the highest sensitivity in their second report Amelard et al. [49].

Even when less informative, there are some results in Table 11 that are worth discussing. Do et al. (2014) [68] achieved promising results on a dataset of 81 images from NSC in Singapore (private dataset) and later in Do et al. (2018) [52] they extended their dataset to 184 images. Subsequently, they tested their model using the MED-NODE dataset and achieved 96.36% accuracy. However, they did not provide the size of their test set. Therefore, we can not guarantee the reliability of their model. On the other hand, Han et al. [25] collected a training dataset consisting of 176,275 images from multiple public datasets, including MED-NODE. After training, they tested the performance of their model with a new test dataset of 8345 images collected from the Asan and Dermofit datasets. Han et al. [25] achieved a sensitivity and a specificity of 91% and 90.4%, respectively, on melanoma binary classification. We believe that Han et al.’s [25] classifier could be highly trustworthy because of the quantity of training and test data they used. Their model performance was tested with a totally different dataset from their training set, which is considered to be the gold standard benchmark in the evaluation of classifiers. Fujisawa et al. [28] used 4867 trained clinical images from the University of Tsukuba (private dataset) with multiple classes and achieved an overall accuracy of 76.5%. They also reported sensitivity and specificity values for malignant and/or benign lesions of 93.4% and 89.5%, respectively. In another study by Pomponiu et al. [40], the authors trained a binary classifier (benign and melanoma) on 399 images picked out from the DermIs and DermQuest datasets and achieved an accuracy of 93.6%, a sensitivity of 95.1%, and a specificity of 92.1%. Additionally, Cavalcanti and Scharcanski [44] used 220 images from the Dermnet and DermQuest and Choudhury et al. [69] used 75 images from the DermNet Nz dataset. Both authors achieved excellent results, as can be observed in Table 11, but the number of training and test images is quite limited. Also, a subset of the DermIs dataset (397 images) was used to train on two different models by Khan et al. [41]. Khan et al. [41] achieved very good results using SVM and DT classifiers separately. But, as we mentioned earlier, their test dataset was too small to be reliable. Meanwhile, Shihab et al. [27] trained a CNN model on the entire DermQuest dataset with 22,080 images and obtained very good results in categorizing malignant and benign lesions. Moreover, their dataset is publicly available, and the number of images used is sufficient. Therefore, the reliability of Shihab et al.’s [27] research would not be questionable. Al-Hammouri et al. [50] trained an extreme learning machine (ELM) with 11 features manually extracted from 200 images (from MED-NODE and Skin Vision datasets) and achieved a sensitivity of 93.9% and a specificity of 100%, outperforming SVM, KNN (k = 5), and RF. In another study, Aggarwal and Papay [38] used 877 images with melanoma and BCC diagnosis and divided them into training, test, and validation sets. The goal of their research was to compare the results of deep CNN classification models trained on artificially darkened skin areas in patients with light-colored skin. Their experiment demonstrated that darkening the skin area in training and validation images can improve the performance of the model. Finally, Yang et al. [26] proposed a pipeline (self-placed balance learning) to deal with the class imbalance in datasets based on extracting features by a CNN and using a weighted SVM as the classifier. Moreover, they employed penalty weight updating and curriculum reconstruction strategies to ensure that the model learns a balanced representation in each self-paced learning procedure.

## 5. Conclusions and Discussion

Computational methods for automated PSL detection can be of great help when assisting dermatologists in the early diagnosis of skin cancer and specifically among computational methods, machine learning has proven to be very effective to aid general practitioners to spot high-risk moles when standard, off-the-shelf cameras are used. In this paper, we reviewed 51 studies that have attempted to detect melanoma using machine learning methods over the past decade, with a focus on the use of clinical datasets, i.e., datasets using standard camera images, as opposed to exploiting more specific tools (dermoscopes) to evaluate suspicious lesions.

Firstly, all the clinical datasets used by the authors have been presented and analyzed. The majority of the clinical datasets in the reviewed state-of-the-art papers were unbalanced, relatively small, or unavailable for public use. This issue can affect the performance of the PSL classifiers negatively since all datasets have a reduced percentage of melanoma and numerous benign lesions, and a large number of articles use accuracy as a quantitative metric of performance measure.

In addition, when describing the experiments, most papers did not divide the dataset into further subsets or did not provide any information regarding their test sets (whether they were different from the validation set or not). Surprisingly, in most cases, all of the data are used for training, and then the same data are used to provide an accuracy estimate that is assumed to show the performance of the system. This is not good practice in machine learning and does not provide a measure of the real performance of the model when faced with a dataset other than that with which it has been trained. To prevent over-fitting, good research practice should include separate training, validation, and testing datasets. This is very important for understanding how well the ML model is generalizing the problem. Every classifier tries to memorize the training set, and this is even more true when the amount of data we use to train the model is small and does not allow the classifier to generalize the problem. For this reason, it is very important to allow the classifier to generalize to new data, and this is not possible with only training and a validation set. Every time a researcher makes a decision about how to change the classifier’s parameters to improve its performance (hyper-parameter tuning), they are actually providing information to the classifier about the validation set. So, after several experiments, the validation data bleed into the training data. A possible way to solve this is to have more annotated data (a test set, in addition to the validation set already used), which is then hidden during the training process, and never examined until a final decision has been made about the tuning of the classifier. Then, the researchers are ready to use a test set to measure the actual error and the real performance of the model. Therefore, we conclude that this flaw in the reviewed articles may impact the performance comparison of different models.

Secondly, we carried out the process of reviewing the implementation of automated skin lesion detection, step by step, and explained each subprocess in detail. The first step of building an automated machine learning model is pre-processing the images. We divided all the pre-processing approaches utilized in the reviewed papers into four categories: illumination correction, artifact removal, image cropping, and data augmentation. It could be observed that the artifact removal approaches were not effective for all cases. We have argued that artifact removal tasks are not absolutely necessary and can be avoided in some cases, depending on the nature of the dataset. In other cases, using an artifact removal method can be beneficial to the general performance of the model. But, until now, available artifact removal approaches have some flaws, thus they should be used with caution. In addition, illumination correction and image cropping can be implemented where they are needed. On the other hand, data augmentation is essential when we are dealing with small and unbalanced datasets.

Segmentation is often the second step in developing automated computational systems for diagnosing skin lesions. Segmentation is also one of the most challenging parts of the process. Among all the applied segmenting methods in the state-of-the-art articles, Otsu’s threshold-based method and K-means (with K = 2) clustering algorithm were the most popular. On the other hand, some authors proposed new segmentation approaches in their works that were mostly based on pre-existing segmentation methods. However, since most of them did not provide a trustworthy evaluation metric, we were not able to provide a quantitative comparison.

In the third step, skin lesion features can be extracted (from the actual images or segmented regions) in order to obtain information for classification. The reviewed papers extracted features either manually (based on the ABCD rule + texture criteria) or automatically (using CNNs). The authors extracted various combinations of attributes such as their asymmetry, border, color, and texture hand-crafted features. Most of the reviewed articles extracted feature descriptors manually based on the ABCD rule. However, hand-crafted features usually require a feature selection and normalization step to improve the performance of the model. On the other hand, papers that applied CNN leave the feature extraction step to be performed automatically by the network. In recent years, CNNs have grown in popularity as a means of automated feature extraction, and as can be seen from the Section 4.4, papers that used automatic methods demonstrated very good performance. In addition, automated features take less time and effort, which makes them more convenient to use. And for skin lesion diagnosis, the skin area surrounding the lesion can provide further information regarding the type of mole. In CNNs, these skin features are automatically taken into account, while hand-crafted features are usually extracted from segmented masks without considering the tissue around the lesion.

The last step of developing an automated PSL diagnosis system is classification. CNNs and SVMs were the most commonly used classifiers in the reviewed papers and achieved better results than the other methods. Also, papers that extracted features manually used trained SVMs to classify the lesions based on their hand-crafted features, while CNNs were used directly on the dataset images in the other studies. Additionally, we saw that during the past few years, CNNs were often the preferred choice over SVMs for feature extraction, due to their ease of use and precision in learning features from the data. Moreover, as we observed in the results section, the reviewed state-of-the-art articles that trained CNNs for classification showed slightly better performance than other methods. Since deep models are currently progressing rapidly, it is expected that more trustworthy models with better performance will appear in the future. Additionally, with the prompt appearance of skin lesion databases, we expect to see more deep models with multi-class classification abilities, providing accurate risk scores and lesion assessments for different types of skin cancer. However, we must mention that CNNs only perform well when they are trained on a corpus of images large enough to yield sufficient samples for all classes. Because the number of melanoma samples is usually limited, researchers who work on melanoma classification may still prefer to use SVMs with hand-crafted features over CNNs, since they provide better generalization with limited data.

On the other hand, in dermatological examinations, skin lesions are usually evaluated in comparison to their neighboring lesions for suspiciousness in order to determine whether further examination or biopsy is necessary. Therefore, to determine if a lesion is malignant or benign, it is important to examine other lesions of the patient as well. In addition, malignant lesions often grow and change over time. Hence, keeping track of lesion changes is also a crucial index in diagnosing PSLs. However, there has been very little work conducted in the area of detecting PSLs using clinical regional images.

Computer-aided diagnosis systems for skin lesions have improved noticeably during the past decade. With the progress of deep neural networks and the appearance of large dermoscopic datasets, CAD systems are now able to diagnose PSLs with high reliability. However, these models are still not capable of replacing professional dermatologists because, first of all, they do not cover all lesion diagnosis criteria, and secondly, there are still some limitations when it comes to imaging the lesions.

As we discussed in Section 4.5, malignant lesions usually grow and evolve over time. Therefore, dermatologists track suspicious lesions over time by having regular examinations. To our knowledge, there is no reliable work conducted on the automatic diagnosis of skin cancer that takes change detection into account. Also, a change in a lesion may be too small to be detected by the naked eye. Hence, having an automated change detection system can also support dermatologists in the early detection of skin cancer and melanoma.

Another important step in skin lesion diagnosis is full-body examinations. The suspiciousness of a lesion can be ranked based on other lesions present on the body of the patient. A lesion may be considered malignant in one patient and benign in other patients based on the overall type and nature of other lesions present on the skin of the patient. Currently, the majority of available CAD systems are trained on single-lesion images. Moreover, to date, there is no publicly available dataset that contains wide-field images. Having such datasets at hand can result in further progress in automated PSL diagnosis.

One of the most important limitations of skin lesion imaging is the presence of hair and other artifacts on the lesions or their surrounding skin area. As we reviewed in Section 4.2, currently, there are no pre-processing methods that can remove the artifacts effectively. As a result, intelligent classifiers are still not able to diagnose those types of images properly. Another limitation in work conducted on clinical data is the lack of public datasets with sufficient numbers of images and diversity in classes that can be used to train a reliable classifier that would be able to diagnose and differentiate all types of skin cancers.

We believe that overcoming the obstacles mentioned above would result in great progress in automated PSL fields and the development of smart devices that could be used in the early detection of melanoma.

## Figures and Tables

**Table 1:** Comparison of our presented review vs. other recent surveys. The criteria takes into consideration the year scope they covered, imaging modality, existence of an overview of all the clinical datasets, and dedicated explanation of pre-processing, segmentation, feature extraction, and classification tasks.

Content	Presented Survey	Stiff et al.	Wen et al.	Manhas et al.	Bhatt et al.	Jones et al.	Haggenmüller et al.	Dildar et al.
		[12]	[13]	[14]	[15]	[16]	[17]	[18]
Year Scope	2011–2022	-	-	2008–2019	-	2000–2021	2017–2021	2011–2021
Focus on clinical images	Yes	Yes	Yes	No	No	No	Yes	No
Overview of all clinical datasets	Yes	No	Yes	No	No	No	No	No
Pre-processing	Yes	No	No	No	No	Yes	Yes	No
Segmentation	Yes	No	No	Yes	Yes	No	No	No
Feature extraction	Yes	No	No	Yes	Yes	No	No	No
Classification	Yes	Yes	No	Yes	Yes	Yes	Yes	Yes

**Table 2:** Datasets information used by the reviewed articles. Reviewed articles utilized different combinations of the subset images obtained from the multiple datasets. NQ: not quantified by author.

Paper	Dataset Source	Total Images
[24]	Train: Asan, Nromal, Web, MED-NODE	220,680
	Test: Dermofit, SNU	1300, 2201
[25]	Train: Asan, Additional Asan, Atlas, MED-NODE, Hallym	179,027
	Test: Dermofit, Asan	1300, 1276
[26]	SD-198, SD-260	6584, 20,600
[27]	DermQuest	22,080
[28]	Digital clinical images collected at University of Tsukuba	4867
[29,30]	PAD-UFES-20	2298
[31,32]	PAD-UFES-20	2057
[33,34]	Collected by authors	2000, NQ
[35]	PAD-UFES-20	1612
[36]	Dermofit	1300
[37]	DermIs, DermQuest, DanDerm, DrrmNet NZ, DermAtlas	1200
[38]	Hellenic Dermatological Atlas, Dermatology Atlas, DermNet Nz, Interactive dermatology atlas	877
[39]	DermAtlas, DermNet, DermIs, Skin Cancer and Benign Tumor Image Atlas,	408
	YSP Dermatology Image Database, Saúde, skin cancer guide	
[40]	DermIs, DermQuest	399
[41]	DermIs	397
[42]	DermQuest, DanDerm, DermAtlas, DermIs, DermNetNz	370
[43]	dermAtlas, DermNent, DermNet NZ, DermQuest, dermIs,	282
	Dermatology Atlas, National Cancer Institute	
[44]	DermNet, DermQuest	220
[45]	DermIs	207
[46,47,48,49]	HLIF Dataset(Subset of DermIs, DermQuest)	206
[50]	MED-NODE, Skin Vision	200
[51]	DermIs, DermQuest	191
[52]	Train: National Skin Center (NSC) of Singapore	184
	Test: MED-NODE	170
[53]	Derm1O1, DermNet, DermIS, DermQuest	175
[23,36,54,55,56,57,58,59,60,61,62,63,64]	MED-NODE	170
[65]	DermNet	141
[66,67]	DermQuest	126
[68]	National Skin Center (NSC) of Singapore	81
[69]	DermNet NZ	75
[70]	Online clinical images (no source specified)	50
[71]	Digital images collected at the Kaohsiung Medical University Hospital	20
[72]	DermIS, DermQuest	15
[73]	DanDerm, DermIS, DermAtlas, DermNet NZ, DermQuest	NQ

**Table 3:** Pre-processing methods distribution.

Pre-Processing Task	References
Illumination correction	[43,44,46,47,48,49,51,53,54,55,56,59,63,65,66]
Artifact removal	[23,27,39,41,42,43,53,54,55,56,59,63,67,71]
Data augmentation	[28,31,35,36,40,51,55,62,73]
Image cropping	[23,25,28,42,46,47,48,49,69]

**Table 4:** Total number of features extracted by each reviewed article (NQ: not quantified), listed in decreasing order.

References	Total Features
Mukherjee et al. [60]	1875
Albert [62]	1815
Khan et al. [41]	294
Ramezani et al. [43]	187
Jadhav et al. [73]	128
Amelard et al. [48]	124
Do et al. [52]	116
Sabouri et al. [42]	90
Do et al. [68]	80
Amelard et al. [49]	54
Amelard et al. [46]	52
Amelard et al. [47]	51
Glaister et al. [51]	48
Cavalcanti and Scharcanski [44]	48
Oliveira et al. [39]	44
Shalu [59]	24
Munia et al. [58]	23
Castro et al., Krohling et al. [31,32]	22
Pacheco and Krohling, Lima and Krohling [29,30]	21
Al-Hammouri et al. [50]	11
Jafari et al., Jafari et al. [56,63]	10
Pacheco and Krohling [35]	8
Pillay and Viriri [23]	5
Moussa et al. [72]	4
Giotis et al. [54], Pomponiu et al. [40], Choudhury et al. [69], Yao et al. [57]	NQ

**Table 5:** The separate weight factors and scores for ABCD rule of dermatology.

Feature	Weight Factor	Score Range
A	1.3	0–2.6
B	0.1	0–0.8
C	0.5	0.5–3.0
D	0.5	0.5–2.5

**Table 6:** Machine learning methods used by reviewed papers. CLAM: Cluster-based Adoptive Metric classifier.

ML method	References
Convolutional Neural Network (CNN)	[24,25,27,28,29,31,35,36,37,38,55,57,64,67,73]
Support Vector Machine (SVM)	[26,39,41,42,43,46,47,51,52,56,58,68,73]
K-Nearest Neighbor (KNN)	[40,41,42,44,52,57,58,59,72]
Neural Network (NN)	[30,42,50,60,63,70]
Naïve Bayes (NB)	[41,42,54,59]
Random Forest (RF)	[41,44,58,59]
Ensemble Training	[42,62]
CLAM	[54]
Special Common Dictionary Learning (SCD)	[57]

**Table 7:** MED-NODE dataset binary classification results. HC: hand-crafted features, Auto: automatically extracted features.

Reference	ML Method	Feature Method	Feature Size	Accuracy	Sensitivity	Specificity
Albert [62]	Ensemble	Both	1815	91%	89%	93%
Mukherjee et al. [36]	CNN	Auto	-	90.14%	-	-
Jafari et al. [56]	SVM	HC	10	79%	90%	72%
Munia et al. [58]	SVM	HC	23	89.07%	87.14%	91%
Biasi et al. [64]	CNN	Auto	-	89%	-	-
Mukherjee et al. [60]	NN	HC	1875	85.09%	86.2%	85.5%
Do et al. [52]	SVM	HC	116	77%	84%	72%
Yao et al. [57]	SCDL	HC	NQ	80%	83%	82%
Nasr-Esfahani et al. [55]	CNN	Auto	-	81%	81%	80%
Giotis et al. [54]	CLAM	HC	NQ	81%	80%	81%
Pillay and Viriri [23]	SVM	HC	5	74.28%	76%	75.29%
Shalu [59]	RF	HC	24	82.35%	74.28%	88%
Jafari et al. [63]	NN	HC	10	76%	82%	71%

**Table 8:** Dermofit dataset classification results. AUC-ROC: area under the ROC curve.

Reference	ML Method	Accuracy	Sensitivity	Specificity	AUC-ROC
Mukherjee et al. [36] (Binary)	CNN	90.58%	-	-	
Han et al. [25] (Melanoma class)	CNN	-	85.5%	80.7%	88%
Han et al. [25] (Multi-class)	CNN	-	85.1%	81.3%	89%
Han et al. [24] (Binary)	CNN	-	-	-	92.8%
Han et al. [24] (Multi-class)	CNN	56.7%	-	-	93.9%

**Table 9:** HLIF dataset classification with hand-crafted features results.

Reference	ML Method	Feature Set Size	Accuracy	Sensitivity	Specificity
(Amelard et al., 2013) [46]	SVM	52	81.26%	84.04%	79.91%
(Amelard et al., 2012) [47]	SVM	51	**87.36%**	90.76%	**82.76%**
(Amelard et al., 2015) [48]	SVM	124	83.59%	91.01%	73.46%
(Amelard et al., 2012) [49]	SVM	54	86.89%	**91.60%**	80.46%

**Table 10:** PAD-UFES-20 dataset binary (cancer vs. non-cancer) and multi-class (6 classes) classification results. The authors combined patient clinical information (features) with features extracted automatically. B: binary classification, MC: multi-class classification, ML: machine learning method, BCC: balanced accuracy, SE: Sensitivity, SP: specificity, AUC-ROC: area under the ROC curve.

Reference	ML	Data Size	Features	BCC	SE	SP	AUC-ROC
Castro et al. [31] (B)	CNN	2057	22	**92.39%**	94.28%	35.67%	-
Krohling et al. [32] (B)	CNN	2057	3	85.5%	**96.42%**	-	-
Lima and Krohling [30] (MC)	NN	2298	21	80%	-	-	94.1%
Pacheco and Krohling [35] (MC)	CNN	1612	8	75%	78%	**80%**	**95.8%**
Pacheco and Krohling [29] (MC)	CNN	2298	21	77%	-	-	94.4%

**Table 11:** Classification results for reviewed state-of-the-art articles. Binary classification means benign vs. malignant classification except for the papers that the classes are specified in the reference column.

Classification	Reference	ML Method	Feature Extraction Method	Dataset Size	Feature Set Size	Accuracy	Sensitivity	Specificity
**Binary**	Shihab et al. [27]	CNN	Automatic	22,080	-	99.7%	99%	99.4%
	Khan et al. [41]	SVM	Hand-crafted	397	294	96%	97%	96%
	Cavalcanti and Scharcanski [65]	KNN	Hand-crafted	220	48	96.71%	96.26%	97.78%
	Khan et al. [41]	RF	Hand-crafted	397	294	94%	98%	93%
	Al-Hammouri et al. [50]	ANN	Hand-crafted	200	11	97%	93.9%	100%
	Do et al. [68]	SVM	Hand-crafted	81	80	93.61%	96.67%	90.55%
	Pomponiu et al. [40]	KNN	Automatic	399	-	93.64%	95.18%	92.1%
	Do et al. [52]	SVM	Hand-crafted	354	116	90.01%	96.36%	83.84%
	Sabouri et al. [42]	SVM	Hand-crafted	1200	90	-	89.28%	100%
	Moussa et al. [72]	KNN	Hand-crafted	15	4	89%	-	-
	Ramezani et al. [43]	SVM	Hand-crafted	282	187	82.20%	77.02%	86.93%
	Aggarwal and Papay [38] (MEL vs. BCC)	CNN	Automatic	877	-	-	82%	76%
	Glaister et al. [51]	SVM	Hand-crafted	191	48	78.6%	74.2%	83.3%
	Marín et al. [70]	NN	Automatic	50	-	-	76.56%	87.58%
	Fujisawa et al. [28]	CNN	Automatic	4867	-	76.5%	93.4%	89.5%
	Oliveira et al. [39]	SVM	Hand-crafted	408	44	74.36%	-	-
**Multi-Class**	Choudhury et al. [69]	SVM	Hand-crafted	75	-	96.26%	-	-
	Han et al. [25] (melanoma classification)	CNN	Automatic	181,603	-	-	91%	90.4%
	Yang et al. [26] (SD-198)	SVM	Automatic	6,584	-	67.8%	65.7%	-
	Yang et al. [26] (SD-260)	SVM	Automatic	20,600	-	65.1%	48.2%	-

## Data Availability

Not applicable.

## Appendix A. Clinical Datasets

In this section, we will present the clinical skin lesion datasets that are publicly available. These datasets have been used to train and test automated skin cancer detection models in different studies.

The Asan dataset [25] was obtained from the Department of Dermatology at Asan Medical Center. Additionally, the Hallym and SNU datasets [24] were introduced by the same authors. SNU was established two years after Hallym and encompasses images from the Hallym dataset. The Hallym dataset contains 152 images, while the SNU dataset comprises 2201 images. Thumbnails of images from the Asan, Hallym, Severance, and SNU datasets are publicly available. However, access to full-sized images necessitates formal approval from the local data access or originating hospitals.

DermQuest [126] is another online medical atlas of healthcare professionals who specialize in dermatology and dermatologists. This dataset includes 22,080 clinical images that were also reviewed and approved by renowned international editorial boards. Later on, DermQuest fused with another dataset, Derm101, although, unfortunately, both datasets were deactivated in 2019. However, there were two datasets called SD-198 [127] with 6584 images and SD-260 [26] with 20,600 images that were collected from DermQuest but only SD-198 is currently available.

The most popular dataset of clinical images in CAD systems is MED-NODE [128]. This database was created by the Department of Dermatology of the University Medical Center Groningen (UMCG). Initially, the dataset was used to train the MED-NODE computer-aided melanoma detection system [54]. There are 170 non-dermoscopic images, including 70 melanoma and 100 nevi. The image dimensions vary, ranging from 257 × 201 to 1333 × 3177 pixels.

A very recent dataset was published by Papadakis et al. [129]. Data were collected over a 3-year period from the medical records of all patients diagnosed with cutaneous melanoma at a tertiary university hospital in Germany. A digital camera and ruler were used to capture clinical images of patients with histologically confirmed melanomas. In all cases, the lesions were photographed with the same camera at admission using a commercial digital camera with a high resolution (1600 × 1200 pixels) and with a ruler aligned beside the lesion to allow for smooth scaling. Any hairs on the lesion were removed with a razor prior to photography. This dataset includes 156 images.

Other publicly available data sources that were used in the state-of-the-art papers were: Dermatology Information System database (DermIs) [130], Interactive Dermatology Atlas (DermAtlas) [131], DanDerm [132], DermNet NZ [133], Dermofit [134], National Cancer Institute database [135] Dermatology Atlas [136], YSP Dermatology database [137], Skin Cancer and Benign Tumor Image Atlas (Loyola University) [138], Skin Cancer Guide [139], Saúde Total [140], MoleMapper [141], XiangyaDerm [142], and DermNet [143]. In Table A1, we have provided a list of all of the clinical datasets during the past decade along with their current availability status. It is important to note that some of these datasets may not be readily accessible to all parties and may require authorization permits.

**Table A1:** All clinical datasets until 2022.

Dataset	Availability	Dataset Size
Additional Asan	Available	159,477
Asan	Available	120,780
XiangyaDerm	Available	107,565
Severance (test set)	Available	40,331
Derm 101	Available	22,979
DermQuest	Not available	22,080
SD-260	Not available	20,600
Dermnet NZ	Available	20,000
Dermatology Atlas	Available	11,797
dermIs	Available	7172
SD-198	Available	6584
DanDerm	Available	3000
Hellenic Dermatological Atlas	Available	2660
Mole Mapper	Available	2422
PAD-UFES-20	Available	2298
SNU	Available	2201
Dermofit (Edinburgh)	Available	1300
Interactive Dermatology atlas	Available	1000
MED-NODE	Available	170
Pepadakis	Available	156
Hallym	Available	152
YSP Dermatology Image Database	Available	Not clear
